# Single Bead Affinity Detection (SINBAD) for the Analysis of Protein-Protein Interactions

**DOI:** 10.1371/journal.pone.0002061

**Published:** 2008-04-30

**Authors:** Roberta Schulte, Jessica Talamas, Christine Doucet, Martin W. Hetzer

**Affiliations:** Molecular and Cell Biology Laboratory, Salk Institute for Biological Studies, La Jolla, California, United States of America; University of Arkansas, United States of America

## Abstract

We present a miniaturized pull-down method for the detection of protein-protein interactions using standard affinity chromatography reagents. Binding events between different proteins, which are color-coded with quantum dots (QDs), are visualized on single affinity chromatography beads by fluorescence microscopy. The use of QDs for single molecule detection allows the simultaneous analysis of multiple protein-protein binding events and reduces the amount of time and material needed to perform a pull-down experiment.

## Introduction

Pull-down assays using affinity chromatography resin are effective research tools to study protein-protein interactions. The minimal requirement for a pull-down assay is the immobilization of a purified or recombinant protein (the bait) on a resin (i.e. agarose beads), which will be used to capture and ‘pull-down’ a binding partner (the prey). Typical methods for the detection of protein complexes, such as Western blotting and isotope or fluorescent labeling necessitate the use of a relatively large number of beads and high protein concentrations to identify interacting partners. While significant progress has been made in the detection of small numbers of proteins [1]–[3] and in miniaturizing reaction volumes [4], these methods require specific antibodies and usually increase time and cost of an assay. Here we developed an ultra-sensitive and economical method that can be used in conjunction with standard pull-down reagents such as Ni-NTA beads. Improved detection sensitivity is achieved by directly visualizing protein-protein interactions on the surface of a single bead using fluorescence microscopy.

## Results

**Figure 1:**
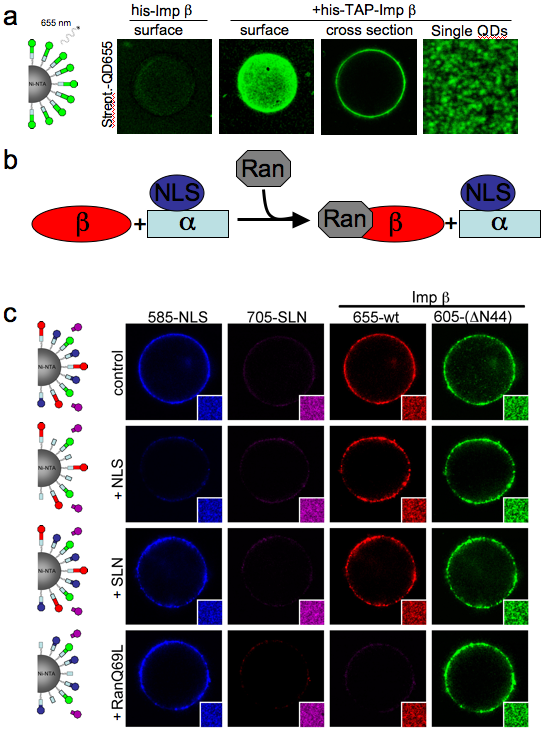
Monitoring protein interactions on a single affinity chromatography bead. (A) 1 µM recombinant his-TAP-tagged Importin β(wt) was immobilized on 5 ul magnetic Ni-NTA beads for 20 min. After the addition of 5 nM CdSe-ZnS core shell QD655 beads were washed and isolated with a magnet. Beads were recovered in 2 ul PBS, mounted on a glass slide and the bead surface was imaged by confocal microscopy. (B) Schematic illustration of the RanGTP (gray) dissociation of Importin β (red) from Importin α (light blue). (c) QD585 cross-linked to NLS peptide (blue), QD705 cross-linked to reverse NLS (SLN) (magenta), streptavidin-coated QD655-TAP-Importin β(wt) (red) and streptavidin-coated QD605-TAP-Importin β(ΔN44) (green) were incubated with his-Importin α coated Ni-NTA beads in the presence of buffer (control), 20 mM NLS or SLN peptides or 10 mM RanQ69L.

As a first step we incubated magnetic Ni-NTA agarose beads with bacterially-expressed nuclear transport receptor Importin β [5] containing an N-terminal poly-histidine (his) tag and tandem-affinity purification (TAP) tag. The TAP-tag encompasses a 38 amino acid streptavidin-binding peptide that binds to streptavidin with a dissociation constant of ∼2 nM. To visualize the immobilized his-TAP-Importin β protein on a single Ni-NTA bead we added commercially available streptavidin-coated QDs with 655nm emission (QD655). The beads were isolated using a magnet, washed, mounted on a glass slide and imaged by confocal microscopy. The surface of his-TAP-Importin β, but not his-Importin β beads, was stained evenly with QD655 (**Figure 1A**), suggesting that the fluorescent nanocrystals specifically bound to the TAP-tagged protein. The distribution of fluorescence intensity among beads was uniform and varied ±∼15% from the population average (**Figure S1A**). At higher magnification individual dots became visible on the bead surface, exhibiting fluorescent intermittency (blinking), which is unequivocal evidence for single molecule detection [6] (**Figure 1A**). Based on the density and the even distribution of QDs on the surface of the beads, we estimate that ∼9.2 million QDs are bound to an average 50 µm bead under saturating conditions (**Figure S1B**). In order to determine the minimal amount of protein that can be detected on one bead, we incubated Ni-NTA resin with decreasing concentrations of his-TAP-Importin β and found that the fluorescence intensity on the Ni-NTA beads decreased from 30 nM to 1 nM (the latter corresponding to ∼20 pg/reaction) before the fluorescence signal reached background levels (i.e. autofluorescence) (**Figure** S**1C**). However, detection sensitivity could be further increased to 10 pM by high resolution imaging of the bead surface. At these concentrations only a few hundred QDs were visible per bead (**Figure** S**1D, E**). This sensitivity was dependent on the use of photo stable QDs because organic fluorophores bleached during repetitive scanning and frame averaging to improve the signal to noise ratio (**Figure** S**1F**).

The ability to detect proteins on a single bead by fluorescence microscopy allowed us to develop SINgle Bead Affinity Detection (SINBAD) as a miniaturized pull-down method. As proof of principle we analyzed the well-characterized association of the nuclear transport receptor Importin α with Importin β and proteins containing a nuclear localization signal (NLS) [7]. Importin α can be released from Importin β by the addition of RanGTP [8] (**Figure 1B**). Since all recombinant proteins in this experiment were his-tagged, we immobilized his-Importin α on CnBr−activated sepharose beads. Next we added QDs conjugated directly to SV40 nuclear localization signal (QD585-NLS), which binds to Importin α, the non-binding mutant NLS (QD705-SLN), wild-type TAP-Importin β labeled with streptavidin-coated QD655 and TAP-Importin βΔN44, a mutant deficient in binding to RanGTP [9], bound to QD605. After the removal of unbound QD-streptavidin conjugates, we tested if the QDs remained stably bound on the proteins by combining the different bead populations and imaging individual beads for 10 min by time-lapse microscopy using three different channels for recording. We did not detect any mixing between the beads suggesting that QDs remained stably bound to the TAP-tag of the immobilized proteins, demonstrating that different proteins can be color-coded using QDs (**Figure S1G**).

All QD-bound proteins, except the negative control QD705-SLN, bound specifically to Importin α resin and could be distinguished on the same bead based on their corresponding QD emission wave lengths (**Figure 1C**). In the presence of excess NLS peptide, but not SLN peptide, QD585-NLS binding to Importin α was inhibited. Upon addition of increasing concentrations of RanQ69L, only wild-type Importin β-QD655 was dissociated, while Importin βΔN44-QD605 remained associated with the bead (**Figure 1C**). This suggests that RanGTP triggered the dissociation of wild-type Importin β from Importin α. Taken together these results demonstrate that QDs can be used to functionally label native proteins and show that detectable pull-down assays can be performed on a single affinity bead. Thus, SINBAD provides a method to perform multiple experiments simultaneously under identical reaction conditions, thereby reducing the time and cost of traditional pull-down assays.

Since millions of binding events can be visualized on the surface of a single bead, the number of beads used per experiment could in principle be reduced to one. To determine the minimal amount of resin that can be reproducibly recovered from a single reaction we made serial dilutions of a 5% slurry of Ni-NTA beads (∼8×10^6^ beads/ml) and then isolated the beads from the diluted suspensions using a magnet. We found that from a 10^6^-fold dilution an average of 7.2±1.05 beads were isolated in 10 independent experiments, which is very close to the expected number of 8 beads (data not shown).

The ability to use less than 10 beads per experiment, combined with the single bead imaging scheme, suggested that the amount of protein used for a pull-down experiment could be reduced significantly.

**Figure 2:**
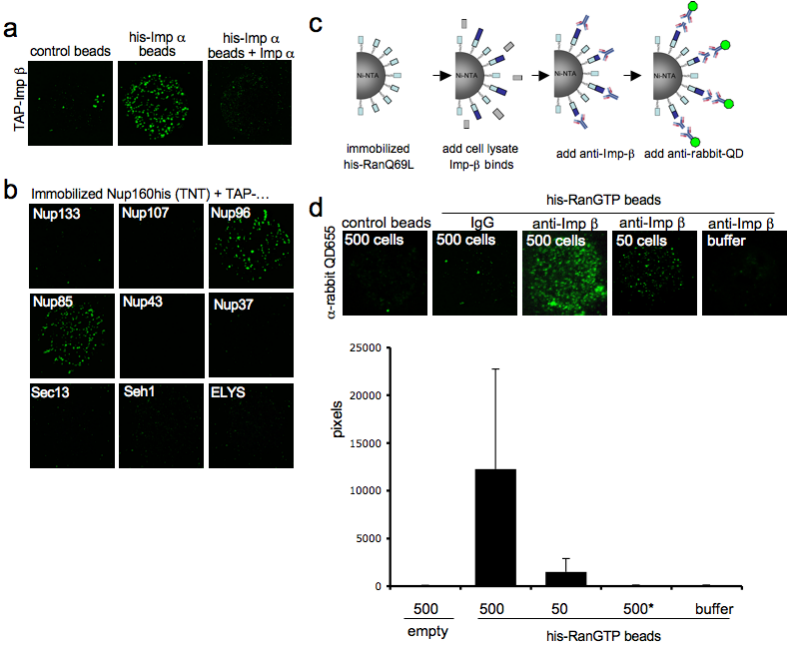
Using SINBAD as pull-down method with increased sensitivity. (A) Approx. 10 Ni-NTA beads were incubated with reticulocyte lysates containing his-Importin α or his- Importin α together with in vitro translated TAP-Importin β(wt) in the presence or absence of excess Importin α. QD655 were added, beads were isolated and imaged. (B) his-Nup160 was translated in reticulocyte lysates and incubated with equal volume (2 ul) of in vitro translated Nup133-TAP, Nup107-TAP, Nup96-TAP, Nup85-TAP, Nup43-TAP, Nup37-TAP, Seh1-TAP, Sec13-TAP and ELYS-TAP for 30 min. Approx. 5 Ni-NTA beads were added together with streptavidin-coated QDs655 and imaged by confocal microscopy on the bead surface. (C) Schematic illustration of SINBAD for the pull-down of endogenous proteins. (D) Hypotonic cell lysates corresponding to 500 or 50 cells were incubated with Ni-NTA beads coated with his-RanQ69L. Bound endogenous Importin β was visualized as described in (C). (E) Random 10 µm^2^ areas were imaged and fluorescence intensity at 655 nm was plotted. n = 20.

Cell-free protein expression systems, such as reticulocyte lysates, are useful for the expression of mammalian proteins, however the protein yield obtained by these methods is usually not sufficient to generate bait columns. To test if SINBAD can be used to perform pull-down assays entirely with in vitro translated protein we expressed his-Importin α and TAP-Importin β (lacking a his-tag) in reticulocyte lysates, each to a yield of ∼2.4 ng/ul as determined by Western blotting (**Figure S2A**). Next, <10 Ni-NTA beads were incubated with 0.1 ul reticulocyte lysates (control beads) or lysates containing translated his-Importin α (his-Imp α beads). After the addition of TAP-Importin β and streptavidin-coated QD655 a fluorescent signal was specifically detected on the his-Imp α beads (**Figure 2A**), indicating that immobilized his-Importin α had pulled down TAP-Importin β thus demonstrating that protein-protein interactions can be analyzed using ∼240 pg of analyte proteins.

Since this binding experiment was performed with nanomolar concentrations (∼27 nM) of Importin α and Importin β, we next asked whether the amount of protein obtained from in vitro-translation is sufficient for competition experiments. We incubated his-Importin α beads with lysates containing TAP-Importin β in the presence of a 10-fold excess of untagged Importin α (300 nM) and found that unlabeled protein indeed competed (his-Imp α beads+Imp α) (**Figure 2A**). These data demonstrate that SINBAD allows the use of standard pull-down reagents to analyze reproducibly protein-protein interactions at nanomolar concentrations.

The ability to utilize in vitro translated polypeptides as ‘bait’ and ‘prey’ proteins with SINBAD offered new opportunities for small-scale protein interaction analyses. Our laboratory studies proteins comprising the nuclear pore complex (NPC) [10], [11], the exclusive site of nucleocytoplasmic transport [12]. The expression of recombinant NPC components remains a technical challenge since many of these ∼30 nucleoporins possess low solubility and molecular weights in excess of 100 kD.

Previously, we found that the nonameric subcomplex Nup107-160 is essential for pore assembly [13]. Attempts to study interactions of its components have met with limited success principally because larger components of the Nup107-160 complex were insoluble when expressed in heterologous systems (data not shown). However, all of the associated nucleoporins including Nup160, one of the largest members of the Nup107-160 subcomplex, could be efficiently translated using reticulocyte lysates (**Figure** S**2B**). Given SINBAD's high sensitivity, we set out to identify the interaction partners of Xenopus Nup160. We first translated His-Nup160 and TAP-tagged versions of Nup133, Nup107, Nup96, Nup85, Nup43, Nup37, Sec13, Seh1 and ELYS *in vitro*. Each of the TAP-tagged nucleoporins was then incubated with equal volumes of his-Nup160 lysate and immobilized on ∼5 Ni-NTA beads. After the addition of streptavidin-coated QDs, beads were isolated and imaged on the surface by confocal microscopy. We found that TAP-Nup96 and TAP-Nup85 specifically bound to the his-Nup160 beads (**Figure 2B**). In a similar manner we imaged direct interactions between Nup96 and Sec13, as well as between Nup107 and Nup133 (data not shown). These findings, which are consistent with the yeast data previously reported [14], confirm that SINBAD is a useful tool to study protein-protein interactions.

Next we tested if SINBAD can be used to decrease the amount of cell lysate required to perform a pull-down an endogenous proteins from cell homogenates. We incubated Ni-NTA beads coated with his-RanQ69L with different volumes of hypotonic 293T cell extracts to pull down endogenous Importin β that was visualized using anti-Importin β and QD-labeled secondary antibodies (**Figure 2C**). We found that the equivalent of 50 cells was sufficient to detect the association of RanQ69L to Importin β (**Figure 2D**). Importantly, by Western blotting at least 7000 cells were required. By comparative Western blotting, we calculated that the amount of Importin β from 50 cells was 35 pg (**Figure S2C** **).** These data are further evidence that SINBAD is a useful method to study protein-protein interactions when cell material is limiting such as with stem cells or rare tissue samples.

**Figure 3:**
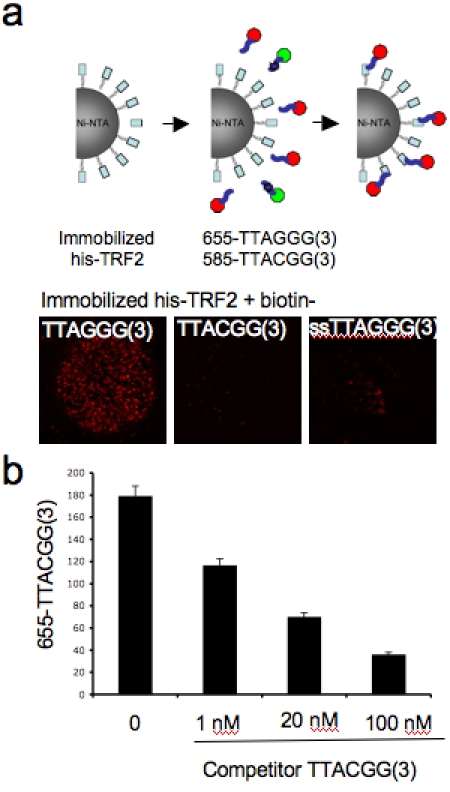
SINBAD for the detection of protein-DNA interactions. (A) his-TRF2 was immobilized on Ni-NTA beads and incubated with hybridized 5′-biotinylated DNA oligos containing three repeats of TTAGGG (red), a mutant TTACGG or ssTTAGG as indicated. Bound DNA was visualized with streptavidin-coated QDs655. (B) <10 his-TRF2 Ni-NTA beads bound to wild-type telomere sequence were incubated with increasing concentrations of competitor DNA oligos. Fluorescence intensity of QD655 was determined from confocal images and plotted.

Since QDs can be used to label DNA [15], we tested if SINBAD allows the study of protein-DNA interactions. As a proof of principle we tested the well-characterized interaction of telomeric DNA sequences with the telomere-binding protein TRF2 [16]. We labeled biotinylated DNA oligos comprising the sequence (TTAGGG)3 with streptavidin-coated QD655 and added recombinant his-TRF2 and Ni-NTA beads. While the wild type sequences efficiently bound to the beads, the mutant (TTACGG)3 lacking the essential G or single stranded DNA did not bind (**Figure 3A**). Similarly when competitor DNA was added binding was abolished (**Figure 3B** **)**. Importantly, these experiments were performed in 10 min and therefore SINBAD provides a rapid alternative to conventional gel-shift assays.

## Discussion

In summary, classical pull-down assays involve the elution and gel-separation of the isolated protein complexes, whereas SINBAD directly visualizes protein-protein interactions on a single bead. The detection limit of SINBAD is 10 pM, which is in the range of other sensitive methods, but does not reach the sensitivity of antibody-or conductivity-based assays [1]–[3]. However, the power of SINBAD does not rely on the detection of single nano-entities. SINBAD is compatible with standard pull-down reagents and does not require the development of miniaturized devices (e.g. involved in field-effect transitions). SINBAD combines the affinity tag platforms (e.g. poly-histidine, GST or TAP), which are widely used for protein purification, with the single molecule detection sensitivity of fluorescence microscopy. Thereby SINBAD overcomes one of the major limitations of pull-down assays, i.e. the requirement of micrograms of proteins.

A similar approach to detect protein-protein interactions by fluorescence microscopy has recently been used to study low affinity interactions between proteins by fluorescent microscopy. This ‘bead halo’ method detects binding of fluorescently labeled proteins in real time under equilibrium binding conditions without washing steps [17]. While the ‘bead halo’ method works well for low affinity interactions, SINBAD successfully combines the ability to study high affinity interactions and to reduce the amount of protein needed for a single experiment. This is relevant since many proteins, which cannot be expressed in *E. coli* or other cellular expression systems, can often be translated at low levels in cell-free systems. Additionally, SINBAD offers a tool to study protein interactions from as little as 50 cells. Second, color-coding of proteins with QDs of different emission wave lengths offers the unique opportunity to monitor multiple binding events in a single experiment on a single bead. We expect that SINBAD can be expanded to other classes of molecules as long as they can be conjugated to QDs. These molecules include but are not limited to RNA, peptides, metabolites or small chemical compounds. Finally, SINBAD provides both a screening platform for identifying new biomolecular interactions as well as an experimental system to characterize the molecular, dynamic and energetic nature of these interactions. Since most laboratories are capable of performing fluorescent microscopy, SINBAD can be easily implemented. Although not tested here, SINBAD should be adaptable for high-throughput setups using large-scale magnetic separation systems.

## Materials and Methods

### DNA constructs and protein expression

His-tagged versions of Importin α, Importin β, Ran wt and RanQ69L were expressed and purified as previously described [18], [19]. Full-length Nup160, Nup107, Nup96 and Sec13 were cloned from Xenopus cDNA library, except Nup133, which was cloned from human cDNA library. The following other Xenopus nucleoporins were PCR amplified from Image clones; Nup37 (#6861092), Nup85 (#7009771), Nup43 (#8321969), Seh1 (#5506962) and ELYS (#5078903). The TAP-tagged versions of the Importin proteins and nucleoporins were generated as follows: the TAP-tag sequence (streptavidin-binding and calmodulin binding peptides) were amplified by PCR from the InterPlay™ vector pCTAP-A (Stratagene) and cloned downstream of the recombination site of the pDEST14 Gateway™ vector (Invitrogen). All proteins were cloned into this vector using gateway cloning per the manufacturers instructions.

Proteins were in vitro transcribed and translated according to the manufacturers instructions using the TNT® T7 coupled reticulocyte lysate system with FluoroTect™ Green_Lys_tRNA (Promega). Protein bands were analyzed by SDS-PAGE and visualized on a fluorescent scanner (Fuji Film FLA-5100, 473 nm). Reactions performed without FluoroTect™ Green_Lys_tRNA were visualized by Western blotting using anti-Calmodulin Binding Peptide antibody (Immunology Consultants Laboratory, Inc.).

To verify expression levels of full-length proteins, fluorescently labeled lysine was added to the translation reactions, proteins were separated by SDS-PAGE and imaged on Odyssey. Core histones were biotinylated as recently described [20].

### SINBAD assay

Except one experiment where we used CnBr-activated agarose (Amersham) (Figure 1b) paramagnetic Ni-NTA beads (Qiagen) were used to immobilize His-tagged proteins (bait). We noticed that some Ni-NTA lots contained less than 1% spherical beads, in contrast to ∼93% in ‘good’ lots, and these beads could not be used for this method. Using this high-resolution imaging approach to detect protein binding we found that under saturating conditions the fluorescence intensity between Ni-NTA beads (Qiagen) varied less than 16% and we found that about 7% of the beads did not bind recombinant protein.

To perform a pull-down assay with ∼10 beads, 5 ul of the 5% slurry bead solution were washed three times in S250 buffer (250 mM sucrose, 50 mM KCl, 2.5 mM MgCl_2_, 20 mM Hepes/KOH pH = 7.4). Beads were incubated with bacterially expressed or in vitro translated proteins for 15–20 min. Unbound protein was removed by three washing steps using S250 buffer. Protein-coated beads were thoroughly resuspended in 1 ml S250.

To test interactions with TAP-tagged proteins 1 ul of the diluted Ni-NTA suspension was incubated with 5-10 ul reticulocyte lysates containing the TAP-tagged recombinant proteins (either bacterially expressed or in vitro translated). After 10 min incubation, beads were isolated, washed three times, recovered in 2 ul S250 and placed on a microscope slide for imaging. Tap-tagged interaction partners were visualized by adding 1 nM streptavidin-coated QDs (wave length used: 525, 565, 585, 605, 655 and 705 nm). QDs were visualized either on the bead surface (e.g. Figure 1) or in cross sections (in this case the bead surface is visible as rim around beads, e.g. Figure 2 and Figure 3c). Image acquisition was performed with a confocal microscope (Leica SP2, Heidelberg). QDs were excited at 405 nm or 488 nm.

### Preparation of DNA beads

MCP1 Plamsid DNA (10 kb) was digested with Not1 and BamH1. After precipitation, 30 ug DNA were labeled in 50 ul fill-in reactions using 5 units/ul Klenow polymerase (Promega), 1 x Klenow buffer, 0.4 mM biotin-UTP and 0.5 mM biotin-ATP for 2 hrs at 37°C. Streptavidin-coated magnetic beads (Dynabeads M280, Invitrogen) were incubated with biotinylated DNA (3 ul beads/ug DNA) in bead buffer (7.5 % PVA, 1M NaCl, 10 mM Tric/HCl pH = 7.6, 1 mM EDTA) at 4°C for 2 hrs, washed three times with bead buffer and stored at 4°C.

#### TRF2 expression and purification

Recombinant his-TRF2 construct was expressed in Baculovirus infected SF9 cells. TRF2 was purified as described previously [16]. Briefly, 48 hours after infection cells were lysed in buffer containing 500 mM NaCl, 20 mM Tris pH7.9 and ‘complete’ protease inhibitors (Roche). TRF2 was isolated using Ni-NTA beads (Invitrogen) and eluted in PBS containing 300 mM NaCl and 250 mM imidizole.

#### Generation of double strand DNA-binding oligos

The DNA-binding and non-binding double strand oligos were generated as reported [16] with slight modifications. The 5′-biolinylated DNA-binding (aatacgactcttagggttagggttaggg) and non-binding (aatacgactcaatacgactcactatagc) oligos were boiled with their complementary DNA for 15 min and slowly returned to room temperature. The resulting double stranded DNA was 100 µM.

## Supporting Information

Figure S1 Characterization of SINBAD method.(A) Experiment was performed as described in Fig. 1A. Fluorescence intensity of QDs on the surface of 43 Ni-NTA beads was determined from confocal images using Image J and plotted. (B) Ni-NTA beads were incubated with increasing concentrations of TAP-Importin β as indicated and number of QDs on the bead surface was determined by confocal microscopy. (C) Ni-NTA beads were incubated with decreasing concentrations of TAP-Importin β as indicated and fluorescence intensity of QDs on the bead surface was determined by confocal microscopy. (D) <10 Ni-NTA beads were incubated with 1 nM, 100 pM or 10 pM TAP-Importin β and bound-protein was visualized by streptavidin-coated QDs655 and imaged. (E) Number of QDs bound to 10 µm^2^ was determined by confocal microscopy and plotted. (F) TAP-Importin β was immobilized on Ni-NTA beads and incubated with TRITC or QD655 -labeled streptavidin and beads were imaged continuously for 10 sec. (G) Three different populations of beads bound to his-TAP-Importin β labeled with either QD585 (green), QD605 (red) or QD655 (blue). After three washing steps to remove unbound QD-streptavidin conjugates, we combined the different bead populations and imaged individual beads for 10 min by time-lapse microscopy (30 frames/min) in three different channels.
(0.27 MB JPG)

Figure S2 Determination of protein levels. (A) Recombinant Importin β either expressed in E. coli or reticulocyte lysates was separated by SDS-PAGE and analyzed by Western blotting using specific anti-Importin b antibodies. (B) TAP-tagged nucleoporins were translated in reticulocyte lysates in the presence of fluorescently labeled lysine, separated by SDS-PAGE and imaged using a fluorescence scanner. (C) Recombinant Importin β and 293T cell lysates were separated by SDS-PAGE and analyzed by Western blotting using specific anti-Importin β antibodies.
(0.10 MB JPG)
