# Supplementary figures and images for: Single Bead Affinity Detection (SINBAD) for the Analysis of Protein-Protein Interactions
Source: PLoS One. 2008 Apr 30;3(4):e2061. doi: 10.1371/journal.pone.0002061 (PMC2329591; doi:10.1371/journal.pone.0002061)

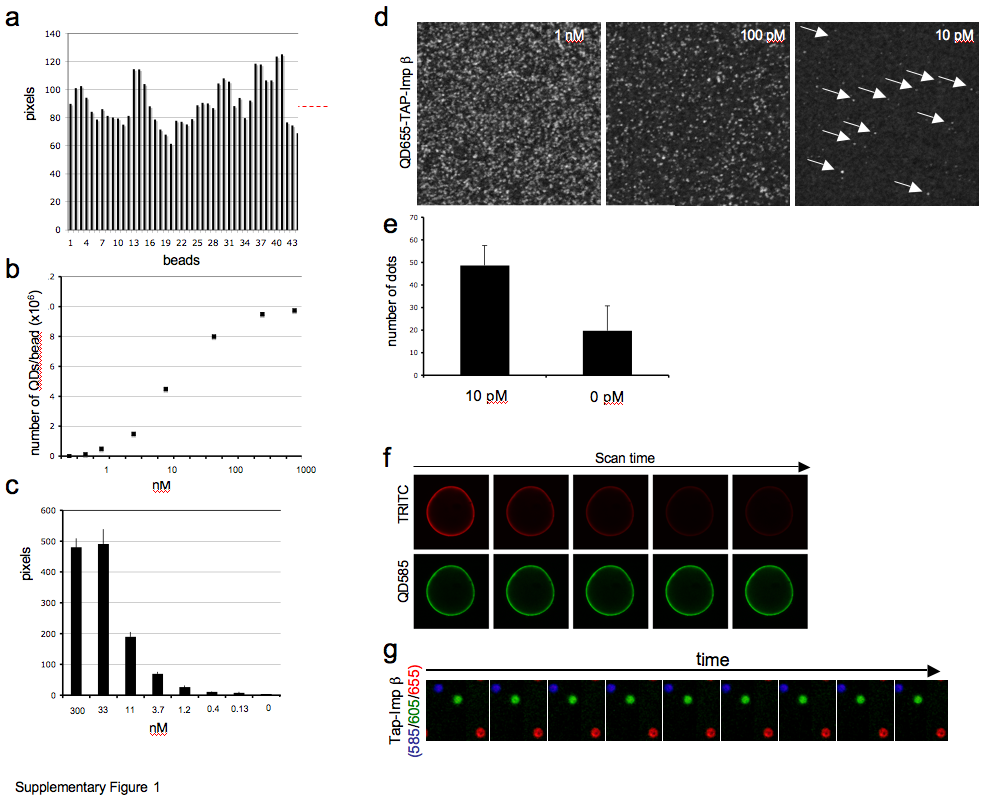

Supplement: Figure S1 — Characterization of SINBAD method.(A) Experiment was performed as described in Fig. 1A. Fluorescence intensity of QDs on the surface of 43 Ni-NTA beads was determined from confocal images using Image J and plotted. (B) Ni-NTA beads were incubated with increasing concentrations of TAP-Importin β as indicated and number of QDs on the bead surface was determined by confocal microscopy. (C) Ni-NTA beads were incubated with decreasing concentrations of TAP-Importin β as indicated and fluorescence intensity of QDs on the bead surface was determined by confocal microscopy. (D) <10 Ni-NTA beads were incubated with 1 nM, 100 pM or 10 pM TAP-Importin β and bound-protein was visualized by streptavidin-coated QDs655 and imaged. (E) Number of QDs bound to 10 µm2 was determined by confocal microscopy and plotted. (F) TAP-Importin β was immobilized on Ni-NTA beads and incubated with TRITC or QD655 -labeled streptavidin and beads were imaged continuously for 10 sec. (G) Three different populations of beads bound to his-TAP-Importin β labeled with either QD585 (green), QD605 (red) or QD655 (blue). After three washing steps to remove unbound QD-streptavidin conjugates, we combined the different bead populations and imaged individual beads for 10 min by time-lapse microscopy (30 frames/min) in three different channels. (0.27 MB JPG) [file pone.0002061.s001.png]

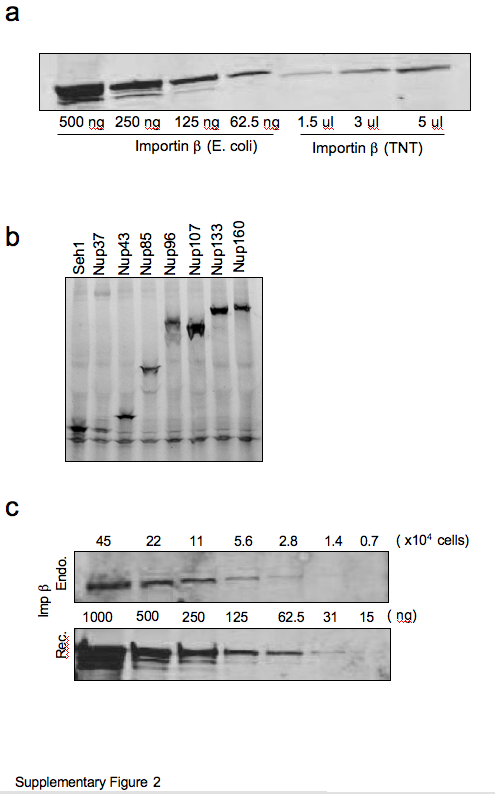

Supplement: Figure S2 — Determination of protein levels. (A) Recombinant Importin β either expressed in E. coli or reticulocyte lysates was separated by SDS-PAGE and analyzed by Western blotting using specific anti-Importin b antibodies. (B) TAP-tagged nucleoporins were translated in reticulocyte lysates in the presence of fluorescently labeled lysine, separated by SDS-PAGE and imaged using a fluorescence scanner. (C) Recombinant Importin β and 293T cell lysates were separated by SDS-PAGE and analyzed by Western blotting using specific anti-Importin β antibodies. (0.10 MB JPG) [file pone.0002061.s002.png]
